# Antibody levels to variant and conserved *Plasmodium falciparum* antigens predict reduction in parasite burden in Malian children

**DOI:** 10.3389/fimmu.2025.1745097

**Published:** 2026-01-13

**Authors:** Delesa Damena, Lauren Dang, Amadou Barry, Jonathan P. Renn, Santara Gaoussou, Almahamoudou Mahamar, Oumar Attaher, Djibrilla Issiaka, Robert D. Morrison, Santosh A. Misal, Alassane Dicko, Patrick E. Duffy, Michal Fried

**Affiliations:** 1 Molecular and Pathogenesis Biomarkers Section, Laboratory of Malaria Immunology and Vaccinology (LMIV), National Institute of Allergy and Infectious Diseases (NIAID), National Institutes of Health (NIH), Bethesda, MD, United States; 2 Biostatistics Research Branch, National Institute of Allergy and Infectious Diseases, National Institutes of Health, Bethesda, MD, United States; 3 Malaria Research and Training Center, University of Sciences, Techniques, and Technologies of Bamako, Bamako, Mali; 4 Pathogenesis and Immunity Section, Laboratory of Malaria Immunology and Vaccinology (LMIV), National Institute of Allergy and Infectious Diseases (NIAID), National Institutes of Health (NIH), Bethesda, MD, United States

**Keywords:** parasite density, phagocytosis, *Plasmodium falciparum*, *Plasmodium falciparum* erythrocyte membrane protein 1 (PfEMP1), *Plasmodium* helical interspersed subtelomeric proteins (PHIST)

## Abstract

**Background:**

Proteins expressed on the surface of *Plasmodium falciparum*-infected erythrocytes (IEs) are targets of immunity. Previous studies described that IEs from young children are more readily recognized than IEs from older children. Here, we aimed to identify targets of naturally acquired antibodies that may play a role in protection from malaria.

**Methods:**

We applied immunoprecipitation followed by mass spectrometry (IP-MS) using plasma samples from susceptible and semi-immune children. We then investigated whether 1) antibody levels to membrane-associated proteins identified by IP-MS, or 2) PfEMP1s expressed in IEs of young children predict reduction in malaria disease.

**Results:**

Significant reduction in risk of high parasite density infection was predicted by high antibody levels to DBLγ11 [OR (95%CI): 0.74 (0.63-0.86)] and DBLζ5 [0.80 (0.69-0.93)], as well as two *Plasmodium* helical interspersed subtelomeric proteins: PF3D7_0201600 [0.81 (0.70-0.94)] and PF3D7_0532300 [0.79 (0.68-0.92)]. Opsonic phagocytosis of DBLγ11 and DBLζ5 coated beads was significantly higher in plasma samples with antibody levels at the top tertile compared to those from lower tertiles (p<0.0001). The Relative phagocytosis index positively correlated with antibody levels, r^2^ = 0.61 (p<0.001) and r^2^ = 0.49 (p<0.001) for DBLζ5 and DBLγ11, respectively.

**Conclusions:**

The study showed that high antibody levels against four proteins significantly reduced the odds of high parasite burden in future infections. Our functional study suggests that opsonic phagocytosis may mediate reduction in parasite density by antibodies to DBLγ11 and DBLζ5.

## Introduction

*Plasmodium falciparum* malaria remains one of the most important public health diseases with an estimated 263 million cases and 597,000 deaths (1). Sub-Saharan African countries bear the greatest malaria burden, accounting for 94% of cases and 95% of deaths (1). In endemic regions, pregnant women and children under 5 years of age are the most vulnerable groups. Recent advances in malaria vaccinology led to the development of two licensed malaria vaccines, RTS, S/AS01, and R21/Matrix-M (1). These vaccines are recommended by WHO for children in moderate-to-high-transmission areas to reduce clinical malaria starting from 5 months of age. These vaccines target circumsporozoite protein (CSP) expressed by the sporozoite stage of *P. falciparum* (Pf) (2). However, escape of a single sporozoite from CSP-specific immune responses is enough to initiate a productive blood-stage infection, which can lead to clinical disease (3). Therefore, blood-stage vaccines may provide a second layer of protection against clinical disease and death, when combined with existing vaccines (4, 5). Previous clinical trials involving merozoite proteins, such as merozoite surface proteins 1 and 3 and apical membrane antigen 1, have demonstrated limited effectiveness in field studies [Reviewed in (6)]. These antigens are polymorphic, and redundant invasion pathways hinder the development of broadly neutralizing immunity. However, RH5, a conserved merozoite protein, has recently received more attention due to its promising efficacy against clinical malaria in children (5).

Pf virulence is associated with intravascular sequestration of parasites, primarily mediated by Pf erythrocyte membrane protein 1 (PfEMP1) (7). PfEMP1s are variant surface proteins with high molecular weight (200‐450 kD) that bind a wide array of host endothelial receptors (8) to support sequestration and avoid parasite clearance by the spleen (9). PfEMP1s are encoded by approximately 60 two‐exon highly variant genes (10). The extracellular domain of PfEMP1 is composed of Duffy binding-like (DBL) and cysteine-rich interdomain regions (CIDR), and relatively conserved Exon II encodes the transmembrane domain (TMD), and an intracellular acidic terminal segment (ATS) (9). Based on multiple sequence alignment, DBL and CIDR domains are divided into six (α, β, γ, δ, ϵ, ξ) and three (α, β, γ) major groups respectively (9). PfEMP1s are classified into four main groups (A, B, C and E) and two intermediate groups (B/A and B/C) based on upstream sequences and chromosome location (9).

Antibodies to PfEMP1s (11) and conserved membrane associated parasite proteins have been associated with protection from malaria (12, 13) in children.

To identify novel parasite antigens recognized by naturally protective immunity, we performed immunoprecipitation (IP) of membrane proteins from lab-adapted clinical isolates using pooled plasma samples from children who participated in a longitudinal birth cohort study, followed by mass spectrometry (MS) analysis. We then tested sera from children enrolled in the longitudinal birth cohort to assess the relationship of antibody levels targeting specific Pf membrane proteins with future malaria outcomes such as reductions in SM risk and parasite burden.

## Materials and methods

### Study population

Children were enrolled between September 2011 and May 2015 into a longitudinal birth cohort in Ouélessébougou, Mali as previously described (14). Malaria transmission in the study area is seasonal, with most infections occurring between July and December.

The study was conducted through active follow-up, which included clinical examinations and monthly malaria parasite blood smear microscopy during the rainy season (July-December), every two months during the dry season (January-June), and at any time the child was sick, up to the age of 5 years. Severe malaria was defined as the presence of parasitaemia in combination with at least one of the following World Health Organization (WHO) criteria for severe malaria: >2 convulsions in the past 24 hours; prostration; hemoglobin <6 g/dl; respiratory distress; coma (Blantyre score <=2).

### Preparation of membrane proteins

Two clinical isolates collected from Malian children enrolled in a longitudinal birth cohort were lab-adapted as previously described (15). Briefly, parasite isolates were maintained at 4% hematocrit in O+ human erythrocyte in complete parasite growth media composed of RPMI-1640 containing 25 mM HEPES, 4 mM L-glutamine, 5 g/L AlbuMAX II, 0.02 g/L hypoxanthine, and 25 μg/mL gentamicin, 10% naïve plasma). Mature parasites (trophozoite/early schizont stage) were enriched to 90% by using discontinuous percoll gradients as described in (16). Membrane proteins were extracted by sequential detergent isolation using 1% triton X-100 and 1% n-Dodecyl-B-D-maltoside(DDM).

### Immuno-precipitation and liquid chromatography-tandem mass spectrometry

Pool plasma sample was added to protein G Mag Sepharose Xtra magnetic beads (Cytiva) and incubated for 60 minutes at room temperature (RT), followed by wash with Phosphate-Buffered Saline with Tween-20 (TBST) buffer followed by cross-linking of bound IgG with BS^3^ (Thermo-Fisher) according to the manufacturer’s protocol. The parasite pellet was diluted to 0.01% DDM. The lysates were cleared by centrifugation at 13,000 g at RT for 3 minutes. The supernatant was incubated with protein G immobilized antibody for 60 minutes at RT under constant agitation. The conjugated beads were washed five times in TBST buffer. Bound proteins were eluted with 2 mM citrate buffer and neutralized with 50 mM Tris-HCl, pH 8. The eluted proteins were processed and analyzed by LC-MS/MS as previously described (17).

### Expression and purification of recombinant proteins

Recombinant proteins were expressed using mammalian cell system methods (18) or *Escherichia coli BL21(DE3)* (19). The protein sequences of the conserved proteins were obtained from the PlasmoDB database, while the sequences and boundaries of the PfEMP1 domains were sourced from our *Var* gene dataset (17). Recombinant proteins were purified using Ni Sepharose Excel (Sytiva) according to the manufacturer’s instructions.

### Multiplex immunoassays

Recombinant proteins and BSA (negative control) were conjugated to Bio-Plex Pro magnetic COOH beads according to the manufacturer’s instructions. The immune assay was performed as previously described (20).

### THP-1 culture and *in vitro* bead phagocytosis assay

THP-1 (ATCC) cell lines were maintained in THP-1 Cell Complete Growth Medium (Innovative) at 37°C in a 5% CO2 incubator. THP-1 cells were tested for surface receptor expression of CD64/FcγRI (Anti-Human CD64 PE, eBioscience) and CD32/FcγRII (Anti-Human CD32 FITC eBioscience), by flow cytometry. *In vitro* bead phagocytosis assay was conducted as previously described (21) except that one million carboxylated fluorescent beads (CFL-5052-2; 5.0-5.9 μm, Spherotech) were covalently coupled with 5 μg of DBLγ11 and DBLζ5. The analysis included randomly selected plasma samples with antibody levels in the highest tertile (N = 60) and bottom two tertiles (N = 20) using the sample function in base R.

### Statistical analysis

Antibody levels were normalized across all plates using anti-His mean fluorescence intensity (MFI), and levels were stratified into tertiles. For the analysis of antibody tertiles as predictors of reduction in parasite density, parasitemia was categorized into five levels based on the distribution of parasite density in the whole cohort. This could be considered as an ordinal measure of parasite burden progression in children. Parasitemia cutoff points were defined based on quartiles of 4,576 blood smears with a count >1 collected from 1758 children with an additional group of children that were not infected during the follow-up period.

To evaluate the association between being in the highest (versus lowest two) antibody tertiles and this ordinal parasitaemia endpoints, an adjacent-category logit (ACL) model was fitted. ACL is a type of ordinal regression model that models the log-odds of being in one category versus the very next highest category of the endpoint (22) in this case category ii v. category i, category iii v. category ii, category iv v. category iii, and category v v. category iv of parasite density. Rather than estimating the association between being in the highest antibody tertile and each of these pairs of adjacent categories separately, the single odds ratio estimated by this model may be interpreted as an average summary measure of all these individual odds ratios. ACL odds ratios that are significantly different than one thus indicates that patients with antibodies in the highest tertile generally have either a significantly higher or lower risk of having a maximum parasite density in a higher category compared to the next lowest category, considering all adjacent categories of this ordinal endpoint.

The ACL model was chosen instead of the more commonly reported proportional odds (PO) model because the ACL model is valid for use with case-control sampled datasets (22). The model was adjusted for age at the time of the visit, whether the blood smear was positive during sample collection, hemoglobin type (categorized as AA, AC, or AS/SC/SS), and weeks from July 1 (the start of the transmission season) at time of sample collection. P-value adjustment was carried out using the Holm method.

To evaluate whether being in the highest antibody tertile for specific candidate antigens predicted reduction in risk of severe malaria in the following malaria season, the odds ratio for being a case (SM) versus a control was compared between those above versus below the highest antibody level threshold. The OR from logistic regression was adjusted for age at the time of the visit, blood smear positivity, hemoglobin type, and weeks from July 1 at the time of sample collection.

The Mann-Whitney U parameter was estimated to compare the age and number of previous infections in individuals within the lowest two antibody tertiles to those in the highest antibody tertile for each antibody. The Mann-Whiteny U test was used to compare the phagocytic activities of samples in the top tertile group with those in the two lower tertiles. Spearman rank correlation was employed to assess the relationship between the phagocytosis index and antibody levels. Analyses were performed using R version 3.6.1 (http://www.R-project.org), and plots were generated with the ggplot2 package.

## Results

### Identifying proteins recognized by naturally acquired antibodies

In high malaria transmission zones, children acquire protective immunity to severe malaria early, however, it takes longer time to acquire immunity associated with a reduction in parasite density (8, 23). We designed paired-IP experiments with two pools of plasma samples, collected before severe malaria (SM) episodes (n=7) or before clinical malaria with high parasite density (n=12), defined as “susceptible” and a pool of plasma samples from the same children collected after the episode, defined as “post-SM/hyperparaitemia”. None of the children had another SM episode, and subsequent infection in the children that had high parasite density infections before, were of lower parasite density.

The two paired plasma pools were used to purify membrane-associated proteins by IP from infected erythrocytes (IEs) of two parasite isolates followed by mass-spectrometry (MS) analysis using our previously described proteomics pipeline designed to identify both conserved and variant parasite proteins (17).

To confirm the proteins detected by the initial IP, we repeated IP-MS analysis with an independent pool of plasma samples collected from 12 children in the same cohort after SM (n=7) or high parasite density infections (n=5). This confirmatory analysis included 4 technical replicates of one of the parasite isolates (**Supplementary Table 1**).

The proteins that were recognized preferentially by post-SM/hyperparasitemia antibodies in at least one of the two paired IP-MS experiments included several new candidate antigens, as well as previously reported proteins such as glutamic acid-rich protein (PfGARP, PF3D7_0113000) (13), enolase (PF3D7_1015900) (24), thioredoxin-like merozoite protein (PF3D7_1104400) (25), and merozoite surface protein 1 (MSP1) (26) (**Supplementary Table 1**).

For the immunosurveillance study, we selected 11 proteins (**Supplementary Table 1**) based on the following criteria: 1) recognized by at least one of the two paired IP-MS (post SM/hyperparasitemia vs. susceptible) experiments; 2) recognized in at least two of the four confirmatory IP-MS experiments; 3) novel proteins with limited or no prior data; and 4) expressed by trophozoites or early schizonts. Of these, 8 were expressed as recombinant proteins, including 4 as full-length proteins and 4 as fragments (**Table 1**). In addition, we included two PfEMP1 domains (3 DBLζ5 variants and 1 DBLγ11 variant) that are commonly expressed by clinical parasite isolates collected from young children at our study site (27). DBLζ5 and DBLγ11 domains were detected in 38.7% and 45.2% of the clinical isolates, respectively as previously described (27). Of the DBLγ11 protein sequences (n=16) expressed in the children’s isolates (27), JOSvar266797_FR4_1–2032 was selected because it was enriched by semi-immune plasma pool in one of the IP experiments (data not shown).

**Table 1:** Recombinant proteins expressed in mammalian (293-expi expression system) and E. coli (BL21-DE3).

Gene ID	Protein name	Molecular weight (KD)	Expressed fragment	Expression system
JOSvar266797_FR4_1-2032	DBLγ11	43	Full length	Mammalian
HB3var07	DBLζ5	49	Full length	Mammalian
PF3D7_0800200	DBLζ5	50	Full length	Mammalian
JOSvar222849_FR6_30-1634	DBLζ5	49.3	Full length	Mammalian
PF3D7_0201800	Knob associated heat shock protein 40 (KAHsp40)	48.3	Full length	Mammalian
PF3D7_0501400	Interspersed repeat antigen (FIRA)_Fragment1	64.1	Fragment [131-711]	Mammalian
	Interspersed repeat antigen (FIRA)_Fragment-2	63.5	Fragment [1141-1720]	Mammalian
PF3D7_1104400	Thioredoxin-like mero protein	49.3	Full length	Mammalian
PF3D7_1015900	Enolase (ENO)	48.7	Full length	Mammalian
PF3D7_0730900	EMP1-trafficking protein (PTP4)-Fragment-1	73.1	Fragment [1-637]	Mammalian
	EMP1-trafficking protein (PTP4)-Fragment-2	64.5	Fragment [1561-2110]	Mammalian
PF3D7_0702400	Small exported membrane protein 1 (SEMP1)	14.2	Full length	E. coli
PF3D7_0201600	PHISTb domain-containing RESA-like protein 1_Fragment-1	10	Fragment [1-110]	Mammalian
	PHISTb domain-containing RESA-like protein-1-Fragment-2	22.5	Fragment [301-485]	Mammalian
PF3D7_0532300	PHISTb, unknown function	31	Fragment [1-270]	Mammalian

To select DBLζ5 that represent domain diversity in the children’s isolates in our dataset, we performed a maximum likelihood phylogenetic tree analysis of the 12 identified domain sequences. We selected three sequences (HB3var07, PF3D7_0800200 and JOSvar222849_FR6_30-1634) that represent each branch of the phylogenetic tree **Supplementary Figure S1**).

### Immuno-surveillance study

#### Characteristics of study participants

Plasma samples for this analysis were collected from children participating in a longitudinal birth cohort in Ouélessébougou, Mali (14). 360 children were included: 99 children with severe malaria (SM) defined as cases, and 261 children without severe malaria matched for age and hemoglobin type, defined as controls. Most samples included in this study were collected during the dry season (N = 341) or the beginning of the rainy season (N = 19). This minimized the likelihood of concurrent infections that might boost antibodies and complicate our efforts to identify protective antibodies. Overall, 10 children were infected at sample collection. All controls included in this study had at least 20 weeks of follow-up during the subsequent malaria transmission season. The mean age of the study participants at the sample collection was 17 and 21 months for cases and controls, respectively (p< 0.001).

#### Association between age, malaria exposure and antibody levels

To evaluate the contribution of factors (such as age and prior malaria infection) to antibody levels, we estimated the Mann-Whitney U parameter comparing these factors for those in the lower two antibody tertiles with those in the highest antibody tertile for each protein (**Table 2**, **Supplementary Figures S2**, **S3**). In this case, the Mann-Whitney U parameter can be interpreted as the probability that a randomly selected individual from the lower two tertiles group would have had fewer prior infections than a randomly selected individual from the highest tertile group. The number of previous infections was significantly lower in children that had antibodies in top tertile (vs. lower two tertiles) against six proteins: DBLγ11 (JOSvar266797_FR4_1-2032), DBLζ5 (JOSvar222849_FR6_30-1634), PF3D7_0532300, F3D7_0201600 (two fragments), PF3D7_1104400 and PF3D7_1015900 (**Table 2**). The number of previous infections was significantly higher in children that had antibody levels in the top tertile (vs lower two tertiles) against four proteins: DBLζ5(PF3D7_0800200), PF3D7_0201800, PF3D7_0501400 (two fragments) and PF3D7_0702400 (**Table 2**). There were no significant associations between age and antibody levels to any of the candidate proteins (**Supplementary Table S2**).

**Table 2:** MW parameter estimates comparing distribution of number of prior infections for children in the highest v. lowest two antibody tertiles for candidate proteins^a^.

Protein	MW Parameter (95% CI)	P-value	Adjusted p-value
DBLγ11_JOSvar266797_FR4_1-2032	0.3 (0.24-0.36)	<0.001	<0.001
DBLζ5_JOSvar222849_FR6_30-1634	0.29 (0.24-0.35)	<0.001	<0.001
DBLζ5_PF3D7_0800200	0.6 (0.53-0.66)	0.003	0.019
DBLζ5_HB3var07	0.46 (0.4-0.52)	0.228	0.912
PF3D7_0201600_fragment 1 (PHISTb)	0.33 (0.27-0.39)	<0.001	<0.001
PF3D7_0201600_fragment 2 (PHISTb)	0.37 (0.31-0.43)	<0.001	<0.001
PF3D7_0532300 (PHISTb)	0.31 (0.26-0.37)	<0.001	<0.001
PF3D7_0730900_fragment1 (PTP4)	0.49 (0.43-0.55)	0.766	1.000
PF3D7_0730900_fragment 2 (PTP4)	0.48 (0.42-0.55)	0.596	1.000
PF3D7_0501400_fragment 1 (FIRA)	0.71 (0.65-0.76)	<0.001	<0.001
PF3D7_0501400_fraggment 2 (FIRA)	0.68 (0.62-0.73)	<0.001	<0.001
PF3D7_1104400 (Thioredoxin-like mero protein)	0.39 (0.33-0.45)	<0.001	0.006
PF3D7_0201800 (KAHsp40)	0.66 (0.6-0.71)	<0.001	<0.001
PF3D7_1015900 (Enolase)	0.41 (0.35-0.47)	0.006	0.030
PF3D7_0702400 (SEMP1)	0.61 (0.55-0.67)	<0.001	0.006

a CI, confidence interval; MW, Mann-Whitney U test; DBL, Duffy binding-like (DBL) domain; PHISTb, Plasmodium helical interspersed subtelomeric proteins; PTP4, Erythrocyte membrane protein 1(EMP1)-trafficking protein 4; FIRA, interspersed repeat antigen; KAHsp40, knob associated heat shock protein 40; SEMP1, small exported membrane protein 1. MW values less than 0.5 indicate fewer prior infections in the highest tertile group, and values greater than 0.5 indicate fewer infections in the lowest tertile group.

#### High antibody levels to surface proteins predict reduction in parasite density

We examined whether antibody responses to the recombinant membrane proteins predict reduction in SM and parasite density in future infections. The analysis included infections that occurred during the immediate malaria transmission season that followed sample collection.

Parasite densities were defined by quartile cutoffs based on 4,576 blood smears with a count >1 collected from the whole cohort of 1758 children. Maximum parasite density was grouped as follows: (i) all 0s (the child did not have a malaria infection during the follow up period), (ii) 1 to 7,475 parasites/μl, (iii) 7,475 to 37,475 parasites/μl, (iv) 37,475 to 112,475 parasites/μl, and (v) 112,475 to 800,000 parasites/μl. We fitted an adjacent category logit model to evaluate whether antibodies in the highest tertile predict reduction in maximum parasite density during the subsequent malaria transmission season. The model was adjusted for age at sample collection, whether blood smear positive at the time of sample collection, hemoglobin type, and weeks from July 1 (the start of the transmission season) at time of sample collection.

**Figure 1:**
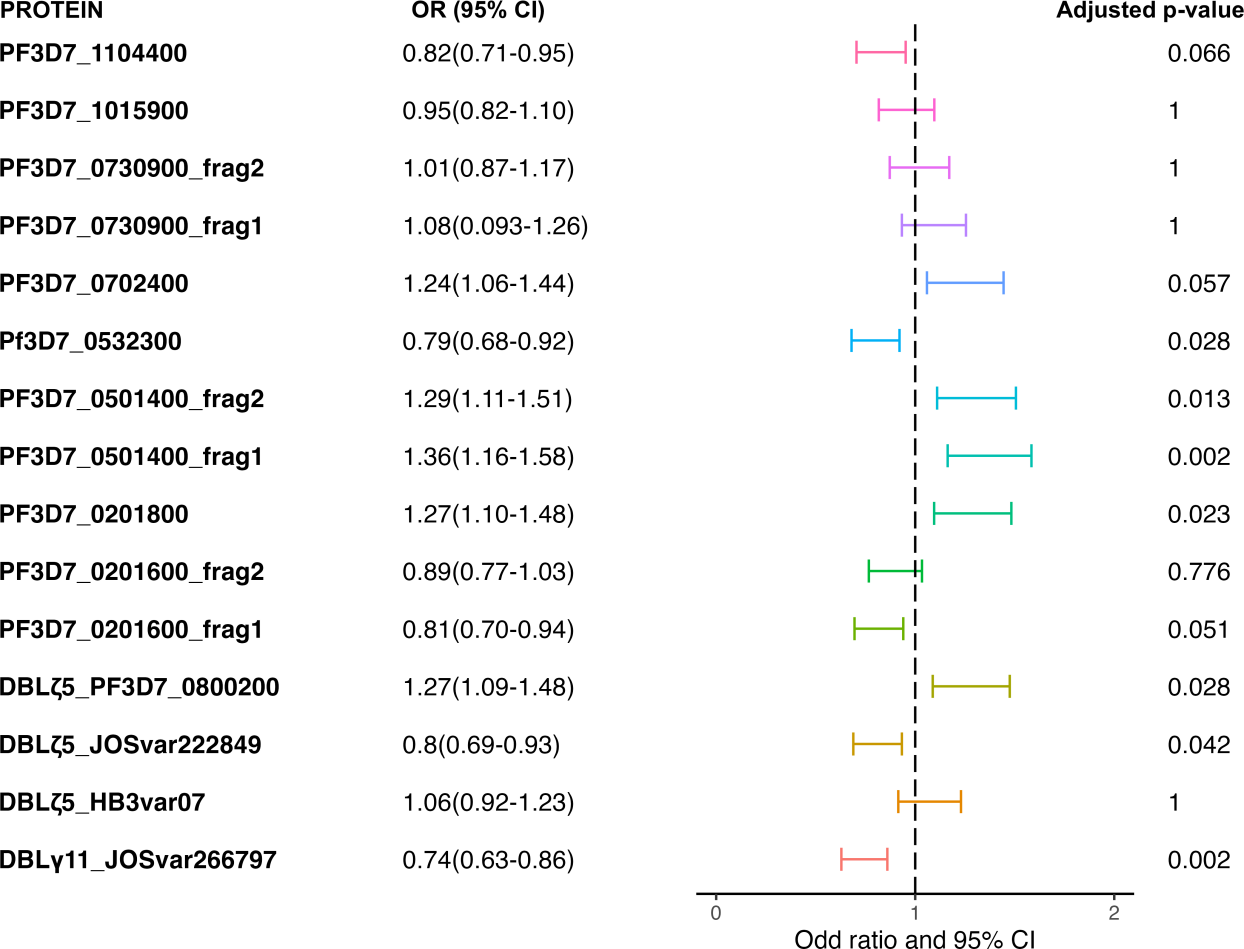
Adjacent-category Logit model analysis of antibody levels as predictors of reduced odds of high parasite density infection during subsequent malaria transmission season. The model was adjusted for age at visit, hemoglobin type, weeks from July 1 at time of sample collection and whether blood smear positive at time of sample collection. Holms-adjusted p values were reported. OR odds ratio; CI, confidence interval.

We observed that high antibody levels (top tertile) to two PfEMP1 domains and two *Plasmodium* helical interspersed sub telomeric (PHISTb) proteins were associated with significantly reduced odds of being in a higher versus lower category of maximum parasite density (**Figure 1**). These included: DBLγ11(JOSvar266797_FR4_1-2032) [OR (95%CI): 0.74 (0.63-0.86), p=0.002]; DBLζ5(JOSvar222849_FR6_30-1634)) [0.80 (0.69-0.93), p = 0.04]; PHISTb domain-containing RESA-like protein 1 [PF3D7_0201600_frag1; 0.81 (0.70-0.94), p = 0.05] and PHISTb [PF3D7_0532300; 0.79 (0.68-0.92), p=0.03). Antibody levels to the proteins examined here were not associated with a reduction in odds of having a severe malaria episode during the immediate malaria transmission season.

#### Phagocytosis activities are correlated to DBLγ11 and DBLζ5 antibody levels

**Figure 2:**
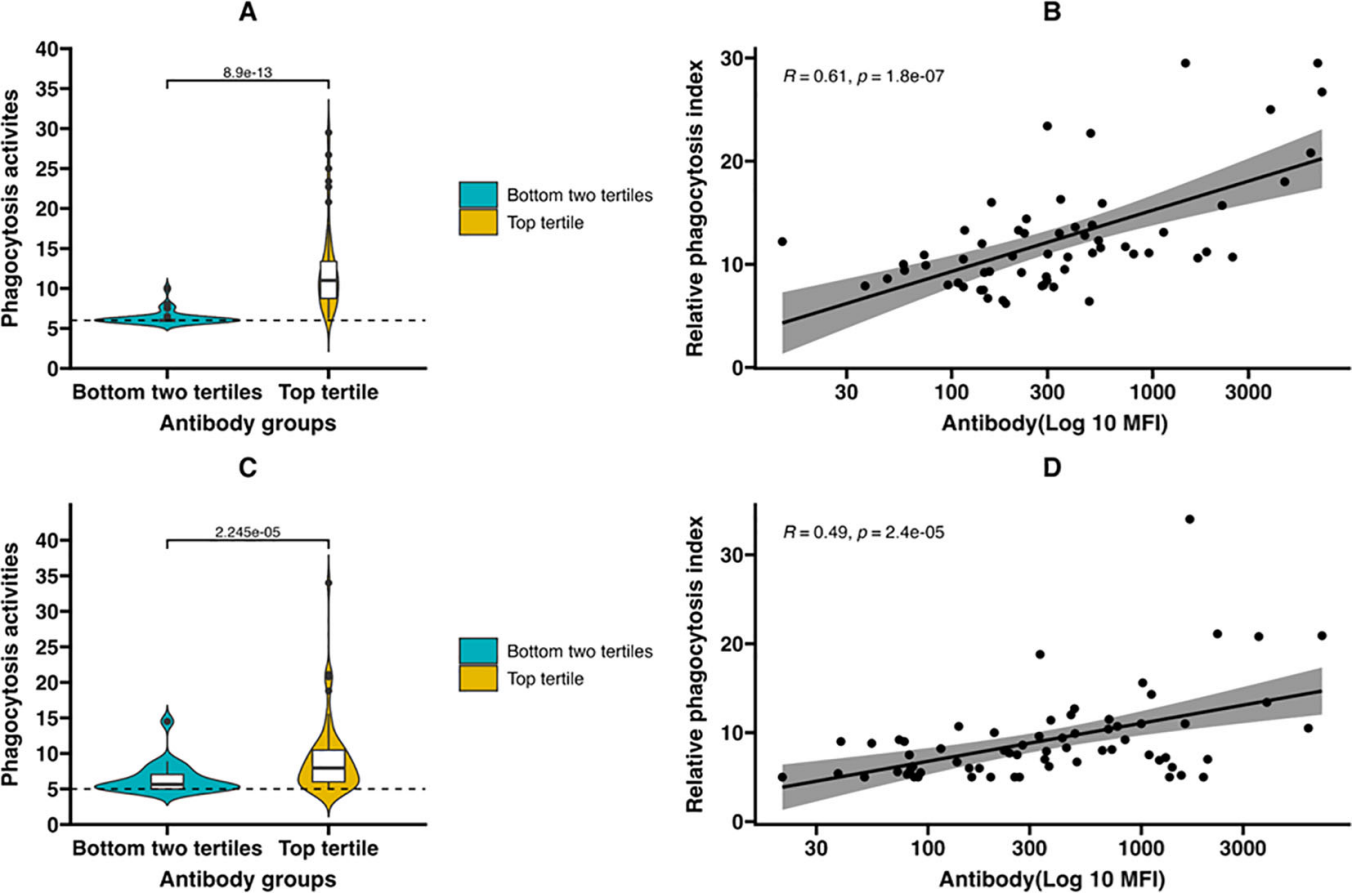
Opsonic phagocytosis of DBLγ11 (JOSvar266797_FR4_1-2032) and DBLζ5 (JOSvar222849_FR6_30-1634) coated beads. Opsonic phagocytosis of plasma samples randomly selected from top tertile (N = 60) and two bottom tertiles (N = 20) for DBLγ11(JOSvar266797_FR4_1-2032) in **(A)** and DBLζ5(JOSvar222849_FR6_30-1634) in **(C)**. Correlation between relative phagocytosis index and antibody titers of plasma samples in top tertile for DBLγ11 in **(B)** and DBLζ5 in **(D)**. Dashed line indicates phagocytosis activity of Naïve pool from US donors.

We investigated whether opsonic phagocytosis contributes to reduction in parasite density by antibodies to DBLγ11 and DBLζ5. Opsonizing activities of beads coated with DBLγ11 and DBLζ5 with randomly selected plasma samples with antibody levels in the highest tertile (N = 60) and bottom two tertiles (N = 20) were compared using human monocytic THP-1 cell line as previously described (21). The opsonic phagocytosis of plasma samples with antibody levels in the top tertile were significantly higher compared to those in the bottom two tertiles for both DBLγ11 (p<0.001) and DBLζ5 (p<0.001; **Figures 2A, C**). The relative opsonic phagocytosis index positively correlated with antibody levels, r=0.61, p<0.001) and r=0.49, p<0.001 for DBLζ5 and DBLγ11, respectively (**Figures 2B, D**).

## Discussions

Antibodies have long been recognized for their crucial role in naturally acquired protective immunity to *P. falciparum* malaria (28). In this study, we utilized plasma sample pools from Malian children before and after severe malaria or high parasite density infection, and proteomic analysis of clinical isolates to select potential membrane-associated target proteins expressed by clinical Pf. This approach enabled us to identify proteins that are differentially recognized by antibodies as children acquire immunity.

PfEMP1s are under conflicting dual evolutionary pressure to maintain host receptor binding capacity while diversifying to evade immune responses (29). Although PfEMP1s are highly variable in sequence, they share a conserved architecture to maintain their binding phenotype (30). Antibodies to PfEMP1s have been shown to be important mediators of protection against malaria (31), but information is limited on the specific PfEMP1 variants targeted by protective immune responses. Our prospective immunosurveillance study showed that high antibody levels to two PfEMP1 domains, DBLγ11 and DBLζ5, significantly decreased the likelihood of a high parasite density infection.

The current finding on DBLγ11 is consistent with our previous study in the same cohort (albeit a different subset of children), showing that high antibody levels to a conserved peptide in DBLγ11 correlated with lower parasite densities in subsequent infections (27). The DBLγ11 peptide previously reported is represented in the DBLγ11 domain studied here: 7/14 and 3/14 amino acids are identical or conserved substitutions respectively. Further analysis is needed to evaluate whether the peptide-containing region is part of a functional epitope.

Earlier research indicated that antibodies to group A or B/A PfEMP1s are rapidly acquired (32, 33) and may predict protection against SM (11). The DBLγ11 and DBLζ5 domains described here could not be assigned to specific PfEMP1 groups because both sequences were identified by RNAseq analysis of clinical isolates that did not include the upstream non-coding sequences used for classification (17).

One important aspect of acquired immunity is the effective control of parasite density during malaria episodes. This can be accomplished by developing antibodies that inhibit the binding of parasites to deep endothelial vasculature, which promote splenic clearance of IEs. Alternatively, antibodies may facilitate clearance of IEs through opsonic phagocytosis or antibody-mediated cytotoxicity or can directly kill or arrest IEs (12, 13, 34, 35).

Here, we observed a significantly higher opsonization (p<0.0001) in plasma samples from children with antibodies in the top tertile for both DBLγ11 and DBLζ5. Phagocytosis index positively correlated with antibody levels for both DBLγ11 (Spearman r=0.49, p<0.001) and DBLζ5 (Spearman r=0.61, p<0.001). These findings support the hypothesis that risk reduction of high density parasitemia in children with high DBLγ11 and DBLζ5 antibody levels may be mediated by opsonic phagocytosis.

In addition to PfEMP1, antibodies targeting two PHIST proteins (PF3D7_0201600 and PF3D7_0532300) were associated with decreased odds of high parasite density infections. PHIST proteins are characterized by a conserved domain of 150 amino acids and represent one of the major families of exported proteins in human malaria species, being highly abundant in the *P. falciparum* proteome (36, 37). PHIST family proteins are localized in various components of IE membranes. PF3D7_0201600 was localized to the surface of the IE (38) while the localization of PF3D7_0532300 is unknown. PHIST proteins can bind to the ATS domain of PfEMP1s and may play a role in cytoadherence (39, 40). A previous study demonstrated that knocking down PHIST PF3D7_0201600 significantly reduced the rate of *var* gene switching and led to increased capacity for chondroitin 4-sulfate-dependent cytoadherence (38). This suggests this PHIST may regulate PfEMP1 expression and the cytoadherence process, but the role of PHIST proteins in immunity remains largely unknown. We speculate that even if the PHIST described here are localized in the inner leaflet of IE membrane, during the later stages of schizogony as IEs membrane become permeable, the proteins can be targeted by antibodies similar to the conserved Pf schizont egress antigen-1 (PfSEA-1) (12). Antibodies to PfSEA, localized to the inner leaflet of the IE membrane, have been shown to reduce parasitaemia and the risk of severe malaria. Further research is needed to characterize the mechanism by which antibodies to the two PHIST proteins reported here mediate protection.

Factors such as prior exposure to malaria and age have been shown to influence antibody levels against different malaria antigens (41, 42). In this study, we investigated the association between age, number of infections prior to blood collection and antibody levels to the 12 selected proteins. The number of prior infections was significantly lower in children with antibody levels in the top tertile versus the two lower tertiles against four proteins that predicted reduction in parasite density, suggesting protective antibodies to these targets are acquired after few infections. Our findings are consistent with previous studies indicating that immunity to clinically important PfEMP1s develops after few infections (7, 32).

Top tertile antibody levels to Thioredoxin-like mero protein (PF3D7_1104400) and Enolase (PF3D7_1015900) were also associated with fewer previous infections. Both Enolase and Thioredoxin-like mero protein have been shown to be localized on the surface of merozoites, and antibodies to these proteins have been associated with protection in animal models (24, 25). Mice immunized with 1) recombinant *P. falciparum* enolase were shown to be partially protected against a lethal challenge with the 17XL strain of the murine malaria parasite *Plasmodium yoelii* (24), and 2) with *P. berghei* ANKA_0942500 protein (the homologue of the thioredoxin-like mero protein) conferred significant protection against lethal infection (25). In the current study, the OR for thioredoxin-like mero protein was 0.82 (95% CI 0.70-0.95), but the association did not remain significant after adjusting for multiple comparisons.

On the other hand, higher antibody levels against four proteins appeared to be associated with an increased likelihood of having a higher density parasite infection (**Figure 1**). For these proteins, children in the top tertile antibodies had significantly higher number of previous infections than children in the bottom two tertiles. We speculate that these antibodies may serve as markers of malaria exposure like apical membrane antigen 1, merozoite surface protein 1, merozoite surface protein 2, and CSP (42).

In this study, we identified new potential candidate antigens as targets of naturally acquired protective immunity. Our prospective immunosurveillance study demonstrated that high antibody levels against four proteins significantly reduced the odds of high parasite density in future infections. Additionally, our functional study suggests antibodies to DBLγ11 and DBLζ5 may mediate risk reduction of high parasite density infection through opsonic phagocytosis. Further research is needed to characterize the PHISTb candidate antigens identified in this study that were associated with reduced risk of high parasite density infection and explore the effector mechanisms mediating their activity. Earlier research indicated that antibodies to group A or B/A PfEMP1s are rapidly acquired (32, 33) and may predict protection against SM (11). The DBLγ11 and DBLζ5 domains described here could not be assigned to specific PfEMP1 groups because both sequences were identified by RNAseq analysis of clinical isolates that did not include the upstream non-coding sequences used for classification. Furthermore, having higher antibodies levels against any of the candidate antigens was not associated with decreased risk of severe malaria in this study. This could be due to the differences in targets of protective antibodies against severe malaria and against parasite burden. Our previous study showed that naturally acquired resistance to severe malaria is not explained by improved control of parasite density (43).

## Data Availability

The original contributions presented in the study are included in the article/**Supplementary Material**. Further inquiries can be directed to the corresponding author.

## Author disclaimer

The contributions of the NIH authors were made as part of their official duties as NIH federal employees, are in compliance with agency policy requirements, and are considered Works of the United States Government. However, the findings and conclusions presented in this paper are those of the authors and do not necessarily reflect the views of the NIH or the U.S. Department of Health and Human Services.
