# Supplementary material for: Antibody levels to variant and conserved Plasmodium falciparum antigens predict reduction in parasite burden in Malian children
Source: Front Immunol. 2026 Jan 13;16:1745097. doi: 10.3389/fimmu.2025.1745097 (PMC12835252; doi:10.3389/fimmu.2025.1745097)
Supplement: Supplementary file 1 [file DataSheet1.pdf]

Supplementary Fig.1. Maximum likelihood phylogenetic analysis of *DBLζ5* domains (N=12) expressed by *P. falciparum* clinical isolates in Malian cohort identified by proteomic methods. Three represented sequences (highlighted in yellow) were selected for immuno- surveillance study.

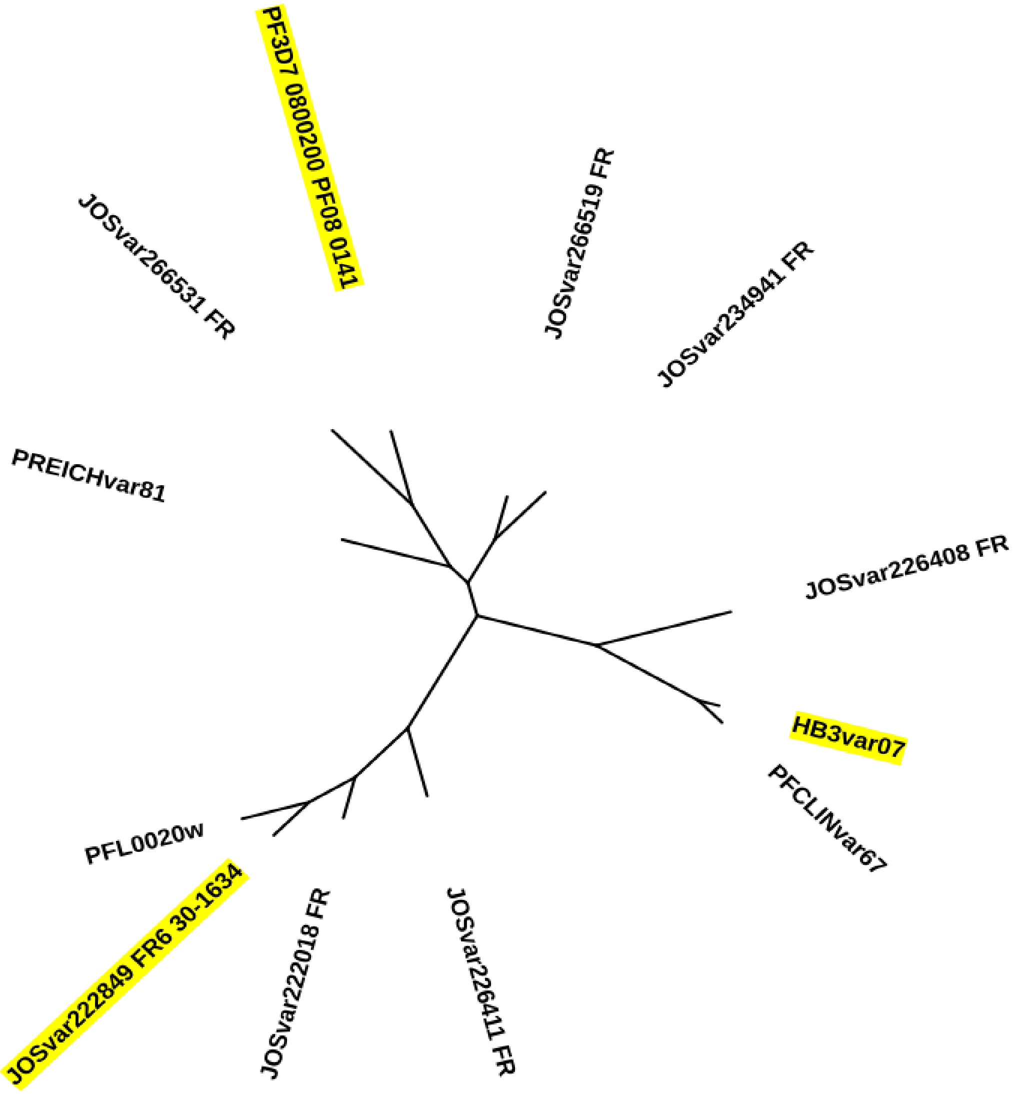

Supplementary Figure 2. Violin plots of number of previous infections comparing those in the lowest two antibody tertiles to those in the highest antibody tertile

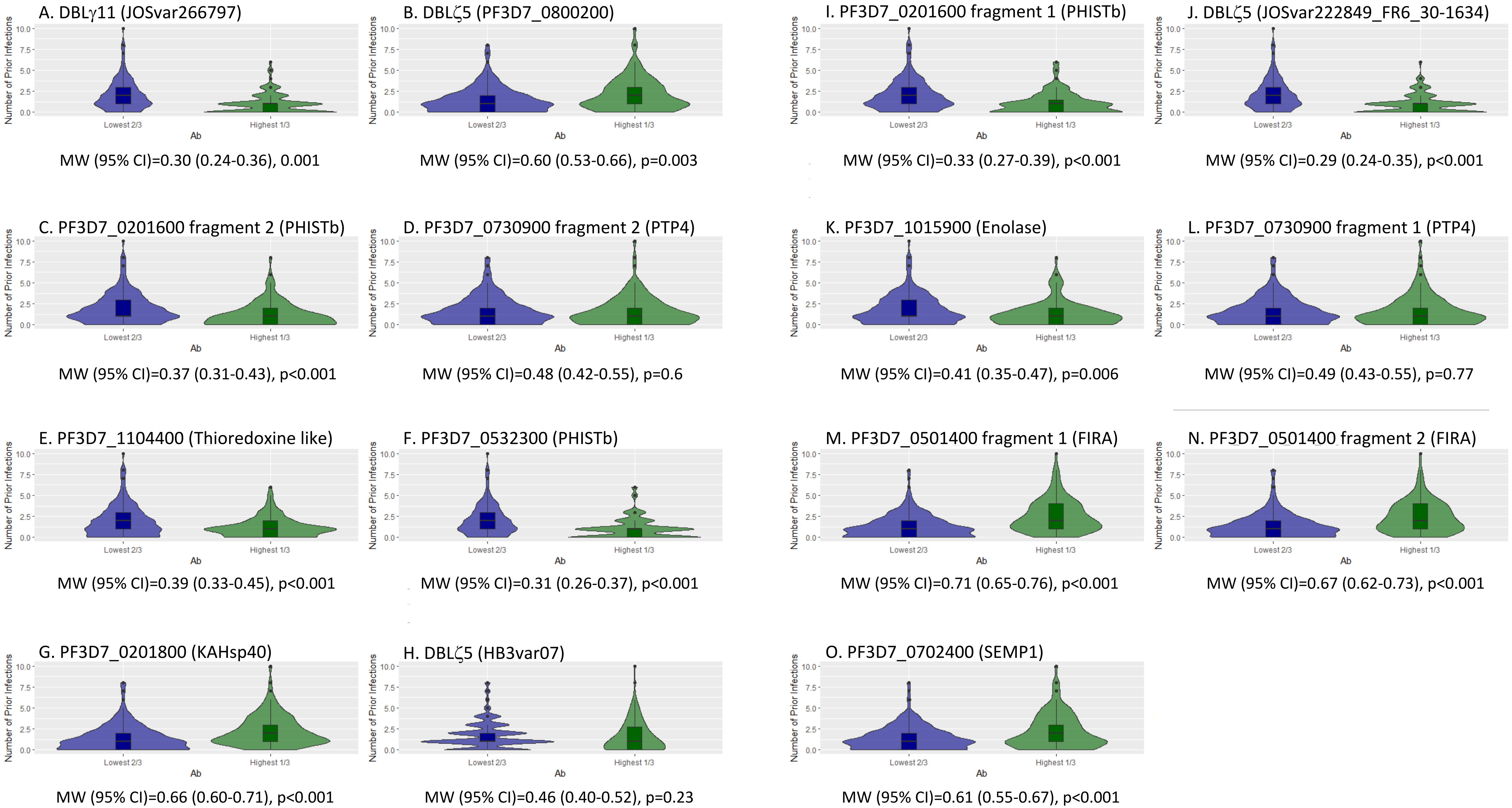

Estimated Mann-Whitney (MW) parameter comparing the number of prior infections for those in the lowest two antibody tertiles (left in blue) to those in the highest antibody tertile (right in green). Values greater than 0.5 indicate that those in the lowest two tertiles group have generally had fewer infections than those in the highest tertile group. Values less than 0.5 indicate that those in the highest tertile group have generally had fewer prior infections than those in the lowest two tertiles group. Values near 0.5 indicate that the numbers of previous infections are similar for the two groups.

Supplementary Figure 3. Violin plots of age comparing those in the lowest two antibody tertiles to those in the highest antibody tertile

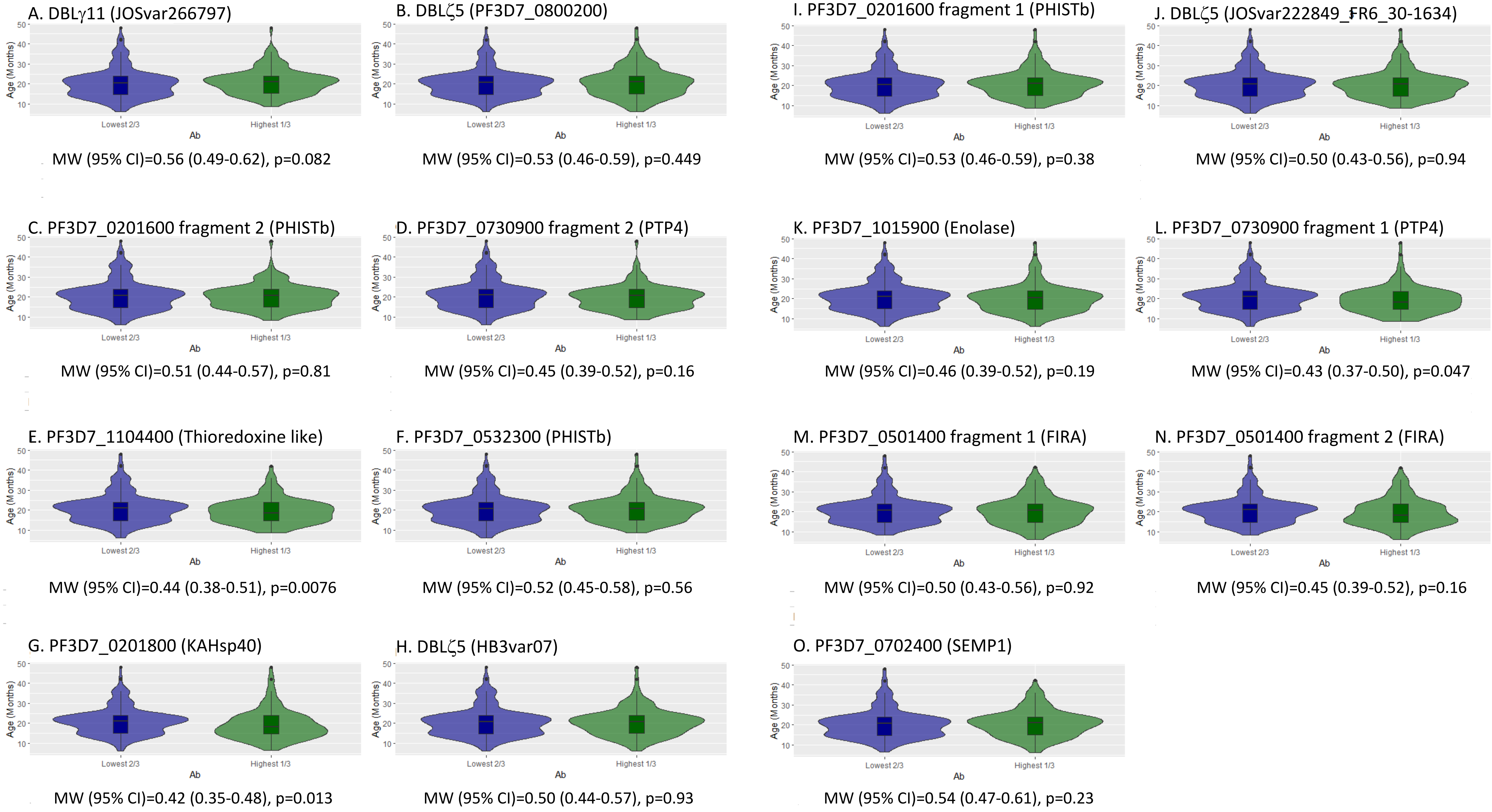

Estimated Mann-Whitney (MW) parameter comparing age for those in the lowest two antibody tertiles (left in blue) to those in the highest antibody tertile (right in green) for each antibody. T Values greater than 0.5 indicate that those in the lowest two tertiles group are generally younger than those in the highest tertile group. Values less than 0.5 indicate that those in the highest tertile group are generally younger than those in the lowest two tertiles group. Values near 0.5 indicate that the ages are similar for the two groups.
