# Supplementary material for: Antibody levels to variant and conserved Plasmodium falciparum antigens predict reduction in parasite burden in Malian children
Source: Front Immunol. 2026 Jan 13;16:1745097. doi: 10.3389/fimmu.2025.1745097 (PMC12835252; doi:10.3389/fimmu.2025.1745097)
Supplement: Supplementary file 2 [file Table1.docx]

**Supplementary Table 1**. Proteins identified by immuno-precipitation followed by mass-spectrometry analysis (IP-MS) experiments using plasma pools collected from semi-immune and susceptible children. Protein abundance was determined by counting the number of peptides detected for each protein. Only proteins with at least two peptides identified in the MS analysis were considered. The proteins highlighted in blue were selected for the immunosurveillance study and expressed, while those highlighted in yellow were selected but could not be expressed.

|  |  | **Paired IP-MS experiment (semi-immune Vs susceptible)** | | | | **Semi-immune IP-MS experiment** | | | |
| --- | --- | --- | --- | --- | --- | --- | --- | --- | --- |
| **Accession** | **Product** | **Isolate 1** | | **Isolate 2** | | **Isolate-1** | | | |
|  |  | **Semi- immune** | **Susceptible** | **semi-immune** | **susceptible** | **Replicate-1** | **Replicate-2** | **Replicate-3** | **Replicate-4** |
| PF3D7_0929400 | high molecular weight rhoptry protein 2 (RhopH2) | 63 | 30 | 78 | 21 | 53 | 9 | 4 | 77 |
| PF3D7_1410400 | rhoptry-associated protein 1 (RAP1) | 43 | 29 | 54 | 25 | 55 | 4 | 0 | 16 |
| PF3D7_0905400 | high molecular weight rhoptry protein 3 (RhopH3) | 30 | 29 | 56 | 28 | 53 | 3 | 2 | 52 |
| PF3D7_0831700 | heat shock protein 70 | 26 | 18 | 32 | 23 | 91 | 18 | 11 | 4 |
| PF3D7_0102200 | ring-infected erythrocyte surface antigen (RESA) | 21 | 18 | 8 | 0 | 42 | 4 | 2 | 18 |
| PF3D7_0501600 | rhoptry-associated protein 2 (RAP2) | 17 | 4 | 19 | 15 | 32 | 5 | 0 | 20 |
| PF3D7_0731600 | acyl-CoA synthetase (ACS5) | 17 | 19 | 17 | 9 | 2 | 3 | 0 | 0 |
| PF3D7_0930300 | merozoite surface protein 1 (MSP1) | 17 | 11 | 10 | 0 | 3 | 15 | 3 | 0 |
| PF3D7_0818900 | heat shock protein 70 (HSP70) | 15 | 10 | 29 | 24 | 73 | 32 | 16 | 2 |
| PF3D7_0501500 | rhoptry-associated protein 3 (RAP3) | 15 | 0 | 17 | 9 | 25 | 0 | 0 | 9 |
| PF3D7_0201800 | knob associated heat shock protein 40 (KAHsp40) | 14 | 0 | 8 | 0 | 12 | 0 | 3 | 0 |
| PF3D7_0302500 | cytoadherence linked asexual protein 3.1 | 13 | 0 | 25 | 11 | 39 | 3 | 10 | 44 |
| PF3D7_0201900 | erythrocyte membrane protein 3 (EMP3) | 13 | 3 | 5 | 2 | 6 | 4 | 13 | 0 |
| PF3D7_1357000 | elongation factor 1-alpha | 12 | 19 | 28 | 16 | 48 | 39 | 76 | 67 |
| PF3D7_1357100 | elongation factor 1-alpha | 12 | 19 | 28 | 16 | 48 | 39 | 76 | 67 |
| PF3D7_0302200 | cytoadherence linked asexual protein 3.2 | 12 | 0 | 27 | 12 | 34 | 0 | 10 | 43 |
| PF3D7_0936800 | Plasmodium exported protein (PHISTc) | 10 | 0 | 10 | 0 | 10 | 0 | 4 | 0 |
| PF3D7_1116800 | heat shock protein 101 (HSP101) | 9 | 20 | 19 | 12 | 23 | 5 | 0 | 8 |
| PF3D7_1104400 | thioredoxin | 7 | 0 | 2 | 0 | 4 | 4 | 4 | 0 |
| PF3D7_0917900 | heat shock protein 70 | 6 | 15 | 17 | 16 | 27 | 15 | 5 | 0 |
| PF3D7_1016300 | glycophorin binding protein (GBP) | 6 | 3 | 16 | 10 | 11 | 16 | 27 | 3 |
| PF3D7_1105100 | histone H2B (H2B) | 6 | 4 | 8 | 5 | 16 | 22 | 31 | 5 |
| PF3D7_0501400 | interspersed repeat antigen (FIRA) | 6 | 0 | 0 | 0 | 0 | 13 | 9 | 0 |
| PF3D7_1252100 | rhoptry neck protein 3 (RON3) | 5 | 0 | 16 | 9 | 7 | 3 | 11 | 0 |
| PF3D7_1105000 | histone H4 (H4) | 5 | 3 | 12 | 7 | 27 | 22 | 50 | 2 |
| PF3D7_0516200 | 40S ribosomal protein S11 | 5 | 4 | 10 | 5 | 7 | 11 | 18 | 3 |
| PF3D7_1033200 | early transcribed membrane protein 10.2 | 5 | 2 | 9 | 9 | 2 | 7 | 18 | 7 |
| PF3D7_0532300 | Plasmodium exported protein (PHISTb) | 5 | 0 | 8 | 0 | 12 | 2 | 0 | 4 |
| PF3D7_1129100 | parasitophorous vacuolar protein 1 (PV1) | 5 | 2 | 8 | 3 | 0 | 3 | 0 | 0 |
| PF3D7_0702400 | small exported membrane protein 1 (SEMP1) | 5 | 4 | 7 | 2 | 13 | 2 | 7 | 8 |
| PF3D7_0320900 | histone H2A.Z | 5 | 4 | 5 | 3 | 2 | 0 | 0 | 6 |
| PF3D7_0919000 | nucleosome assembly protein (NAPS) | 5 | 0 | 2 | 0 | 2 | 0 | 0 | 2 |
| PF3D7_0113000 | glutamic acid-rich protein (GARP) | 5 | 0 | 0 | 0 | 5 | 46 | 26 | 0 |
| PF3D7_1438900 | thioredoxin peroxidase 1 | 4 | 3 | 11 | 5 | 2 | 9 | 17 | 3 |
| PF3D7_0202000 | knob-associated histidine-rich protein (KAHRP) | 4 | 2 | 6 | 0 | 14 | 69 | 76 | 7 |
| PF3D7_1027300 | peroxiredoxin (nPrx) | 4 | 0 | 5 | 0 | 2 | 18 | 88 | 0 |
| PF3D7_0213100 | protein SIS1 (SIS1) | 4 | 0 | 3 | 4 | 9 | 3 | 0 | 3 |
| PF3D7_0501100 | heat shock protein 40 | 4 | 0 | 3 | 0 | 11 | 0 | 0 | 3 |
| PF3D7_0401800 | Plasmodium exported protein (PHISTb) | 4 | 0 | 0 | 0 | 13 | 4 | 23 | 0 |
| PF3D7_1149200 | ring-infected erythrocyte surface antigen | 4 | 3 | 0 | 0 | 8 | 0 | 0 | 0 |
| PF3D7_1302100 | gamete antigen 27/25 | 4 | 6 | 0 | 0 | 6 | 2 | 0 | 10 |
| PF3D7_0814200 | DNA/RNA-binding protein Alba 1 (ALBA1) | 3 | 0 | 11 | 5 | 18 | 10 | 17 | 2 |
| PF3D7_1015900 | enolase (ENO) | 3 | 0 | 10 | 0 | 0 | 0 | 6 | 7 |
| PF3D7_0714000 | histone H2B variant | 3 | 4 | 6 | 5 | 5 | 6 | 20 | 2 |
| PF3D7_1471100 | exported protein 2 (EXP2) | 3 | 0 | 4 | 0 | 2 | 0 | 0 | 5 |
| PF3D7_0601900 | conserved Plasmodium protein unknown function | 3 | 0 | 3 | 0 | 2 | 0 | 0 | 3 |
| PF3D7_1211400 | heat shock protein DNAJ homologue Pfj4 (PfJ4) | 3 | 0 | 3 | 3 | 0 | 3 | 3 | 0 |
| PF3D7_1211800 | polyubiquitin (PfpUB) | 3 | 2 | 3 | 2 | 8 | 2 | 4 | 3 |
| PF3D7_1365900 | ubiquitin-60S ribosomal protein L40 | 3 | 2 | 3 | 2 | 8 | 4 | 5 | 3 |
| PF3D7_0114100 | Pfmc-2TM Maurer's cleft two transmembrane protein | 3 | 0 | 2 | 0 | 0 | 0 | 7 | 2 |
| PF3D7_0222100 | Pfmc-2TM Maurer's cleft two transmembrane protein | 3 | 0 | 2 | 0 | 0 | 2 | 6 | 2 |
| PF3D7_1039700 | Pfmc-2TM Maurer's cleft two transmembrane protein | 3 | 0 | 2 | 0 | 0 | 0 | 5 | 2 |
| PF3D7_1101700 | Pfmc-2TM Maurer's cleft two transmembrane protein | 3 | 0 | 2 | 0 | 0 | 0 | 5 | 2 |
| PF3D7_0935900 | ring-exported protein 1 (REX1) | 3 | 1 | 0 | 0 | 7 | 2 | 0 | 4 |
| PF3D7_1128200 | multiprotein-bridging factor 1 | 3 | 0 | 0 | 0 | 2 | 0 | 0 | 0 |
| PF3D7_1202900 | high mobility group protein B1 (HMGB1) | 3 | 4 | 0 | 0 | 11 | 0 | 0 | 11 |
| PF3D7_1216200 | glycerol-3-phosphate dehydrogenase | 3 | 0 | 0 | 0 | 8 | 0 | 0 | 3 |
| PF3D7_1301400 | Plasmodium exported protein (hyp12) unknown function (HYP12) | 3 | 0 | 0 | 0 | 2 | 0 | 0 | 0 |
| PF3D7_1236100 | \| clustered-asparagine-rich protein | 3 | 0 | 3 | 0 | 0 | 0 | 0 | 0 |
| PF3D7_0731100 | \| EMP1-trafficking protein | 3 | 0 | 0 | 0 | 0 | 0 | 0 | 0 |
| PF3D7_0831400 | \| Plasmodium exported protein unknown function | 3 | 0 | 0 | 0 | 0 | 0 | 0 | 0 |
| PF3D7_1347500 | DNA/RNA-binding protein Alba 4 (ALBA4) | 2 | 0 | 16 | 12 | 10 | 10 | 8 | 5 |
| PF3D7_1224300 | polyadenylate-binding protein | 2 | 0 | 13 | 0 | 2 | 8 | 0 | 0 |
| PF3D7_1437900 | HSP40 | 2 | 0 | 13 | 0 | 0 | 0 | 0 | 0 |
| PF3D7_1246200 | actin I (ACT1) | 2 | 0 | 9 | 2 | 39 | 53 | 15 | 28 |
| PF3D7_1346300 | DNA/RNA-binding protein Alba 2 (ALBA2) | 2 | 0 | 8 | 5 | 2 | 3 | 0 | 3 |
| PF3D7_1232100 | 60 kDa chaperonin (CPN60) | 2 | 0 | 5 | 4 | 3 | 0 | 0 | 0 |
| PF3D7_0517000 | 60S ribosomal protein L12 | 2 | 0 | 4 | 0 | 3 | 5 | 7 | 0 |
| PF3D7_0617800 | histone H2A (H2A) | 2 | 2 | 4 | 2 | 5 | 4 | 8 | 3 |
| PF3D7_1431700 | 60S ribosomal protein L14 | 2 | 0 | 4 | 0 | 4 | 5 | 9 | 0 |
| PF3D7_0730900 | EMP1-trafficking protein (PTP4) | 2 | 0 | 3 | 0 | 11 | 5 | 8 | 0 |
| PF3D7_0404900 | 6-cysteine protein (P41) | 2 | 0 | 2 | 0 | 0 | 3 | 0 | 0 |
| PF3D7_0422300 | alpha tubulin 2 | 2 | 0 | 0 | 0 | 0 | 7 | 8 | 0 |
| PF3D7_0817900 | high mobility group protein B2 (HMGB2) | 2 | 6 | 0 | 0 | 13 | 0 | 0 | 11 |
| PF3D7_1200700 | acyl-CoA synthetase (ACS7) | 2 | 0 | 0 | 0 | 0 | 0 | 0 | 0 |
| PF3D7_1324900 | L-lactate dehydrogenase (LDH) | 2 | 0 | 0 | 0 | 0 | 10 | 7 | 2 |
| PF3D7_1352500 | thioredoxin-related protein | 2 | 0 | 0 | 0 | 0 | 5 | 9 | 0 |
| PF3D7_1412500 | actin II (ACT2) | 2 | 0 | 0 | 0 | 14 | 11 | 3 | 10 |
| PF3D7_0201600 | \| PHISTb domain-containing RESA-like protein 1 | 2 | 0 | 7 | 0 | 0 | 0 | 7 | 2 |
| PF3D7_1133400 | \|apical membrane antigen 1 | 2 | 0 | 0 | 0 | 0 | 0 | 0 | 0 |
| PF3D7_0500800 | mature parasite-infected erythrocyte surface antigen (MESA) | 0 | 0 | 15 | 0 | 12 | 17 | 19 | 0 |
| PF3D7_0501200 | parasite-infected erythrocyte surface protein (PIESP2) | 0 | 0 | 14 | 4 | 0 | 0 | 0 | 0 |
| PF3D7_0424600 | Plasmodium exported protein (PHISTb) unknown function | 0 | 0 | 12 | 3 | 4 | 0 | 0 | 4 |
| PF3D7_1465900 | 40S ribosomal protein S3 | 0 | 0 | 11 | 7 | 6 | 6 | 16 | 0 |
| PF3D7_0322900 | 40S ribosomal protein S3A | 0 | 0 | 10 | 0 | 4 | 10 | 50 | 0 |
| PF3D7_0716800 | eukaryotic translation initiation factor 3 subunit I | 0 | 0 | 10 | 0 | 6 | 0 | 0 | 2 |
| PF3D7_1006200 | DNA/RNA-binding protein Alba 3 (ALBA3) | 0 | 0 | 10 | 7 | 13 | 6 | 16 | 0 |
| PF3D7_0532400 | lysine-rich membrane-associated PHISTb protein (LyMP) | 0 | 0 | 9 | 2 | 2 | 3 | 5 | 0 |
| PF3D7_1010700 | dolichyl-phosphate-mannose protein mannosyltransferase | 0 | 0 | 9 | 0 | 0 | 0 | 0 | 0 |
| PF3D7_1130200 | 60S ribosomal protein P0 (PfP0) | 0 | 0 | 9 | 4 | 8 | 5 | 0 | 0 |
| PF3D7_1242700 | 40S ribosomal protein S17 | 0 | 0 | 9 | 0 | 4 | 7 | 15 | 0 |
| PF3D7_1353100 | Plasmodium exported protein | 0 | 0 | 9 | 0 | 4 | 4 | 18 | 0 |
| PF3D7_0422400 | 40S ribosomal protein S19 (RPS19) | 0 | 0 | 8 | 6 | 5 | 8 | 19 | 2 |
| PF3D7_0520000 | 40S ribosomal protein S9 | 0 | 0 | 8 | 0 | 5 | 8 | 27 | 0 |
| PF3D7_0610400 | histone H3 (H3) | 0 | 0 | 8 | 7 | 13 | 9 | 31 | 5 |
| PF3D7_1302800 | 40S ribosomal protein S7 | 0 | 0 | 8 | 0 | 2 | 10 | 22 | 0 |
| PF3D7_1460700 | 60S ribosomal protein L27 (RPL27) | 0 | 0 | 8 | 2 | 3 | 11 | 34 | 0 |
| PF3D7_0501000 | Plasmodium exported protein | 0 | 0 | 7 | 0 | 0 | 3 | 7 | 0 |
| PF3D7_0519400 | 40S ribosomal protein S24 (RPS24) | 0 | 0 | 7 | 0 | 2 | 10 | 19 | 0 |
| PF3D7_0629200 | DnaJ protein | 0 | 0 | 7 | 0 | 0 | 0 | 0 | 3 |
| PF3D7_0814000 | 60S ribosomal protein L13-2 | 0 | 0 | 7 | 0 | 3 | 10 | 29 | 0 |
| PF3D7_1126200 | 40S ribosomal protein S18 | 0 | 0 | 7 | 0 | 4 | 6 | 14 | 0 |
| PF3D7_1145400 | dynamin-like protein (DYN1) | 0 | 0 | 7 | 2 | 2 | 0 | 0 | 0 |
| PF3D7_1201000 | Plasmodium exported protein (PHISTb) | 0 | 0 | 7 | 0 | 0 | 5 | 0 | 0 |
| PF3D7_1220900 | heterochromatin protein 1 (HP1) | 0 | 0 | 7 | 5 | 0 | 2 | 0 | 0 |
| PF3D7_1408600 | 40S ribosomal protein S8e | 0 | 0 | 7 | 0 | 5 | 9 | 49 | 0 |
| PF3D7_1421200 | 40S ribosomal protein S25 (RPS25) | 0 | 0 | 7 | 0 | 2 | 4 | 13 | 0 |
| PF3D7_1441200 | 60S ribosomal protein L1 | 0 | 0 | 7 | 0 | 0 | 0 | 3 | 0 |
| PF3D7_0208800 | conserved Plasmodium protein | 0 | 0 | 6 | 0 | 0 | 2 | 0 | 0 |
| PF3D7_0315100 | eukaryotic translation initiation factor 4E (eIF4E) | 0 | 0 | 6 | 0 | 0 | 0 | 0 | 0 |
| PF3D7_0316800 | 40S ribosomal protein S15A | 0 | 0 | 6 | 0 | 0 | 5 | 13 | 0 |
| PF3D7_0317600 | 40S ribosomal protein S11 | 0 | 0 | 6 | 0 | 2 | 7 | 16 | 0 |
| PF3D7_0617900 | histone H3 variant | 0 | 0 | 6 | 5 | 12 | 8 | 26 | 0 |
| PF3D7_0813900 | 40S ribosomal protein S16 | 0 | 0 | 6 | 0 | 4 | 5 | 22 | 0 |
| PF3D7_0923900 | RNA-binding protein | 0 | 0 | 6 | 0 | 0 | 2 | 5 | 0 |
| PF3D7_1117700 | GTP-binding nuclear protein RAN/TC4 (RAN) | 0 | 0 | 6 | 0 | 9 | 11 | 13 | 2 |
| PF3D7_1447000 | 40S ribosomal protein S5 | 0 | 0 | 6 | 6 | 3 | 9 | 19 | 0 |
| PF3D7_0316700 | protein YOP1 | 0 | 0 | 5 | 0 | 0 | 0 | 7 | 0 |
| PF3D7_0719700 | 40S ribosomal protein S10 | 0 | 0 | 5 | 0 | 0 | 2 | 5 | 0 |
| PF3D7_0813300 | conserved Plasmodium protein | 0 | 0 | 5 | 0 | 10 | 5 | 0 | 0 |
| PF3D7_0818200 | 14-3-3 protein | 0 | 0 | 5 | 4 | 9 | 9 | 7 | 5 |
| PF3D7_0827900 | protein disulfide isomerase (PDI8) | 0 | 0 | 5 | 0 | 0 | 0 | 0 | 0 |
| PF3D7_0935800 | cytoadherence linked asexual protein 9 (CLAG9) | 0 | 0 | 5 | 0 | 0 | 0 | 8 | 0 |
| PF3D7_1134100 | protein disulfide isomerase | 0 | 0 | 5 | 0 | 0 | 0 | 0 | 0 |
| PF3D7_1301700 | CX3CL1-binding protein 2 (GEXP07) | 0 | 0 | 5 | 3 | 2 | 0 | 2 | 0 |
| PF3D7_1323100 | 60S ribosomal protein L6 putative | 0 | 0 | 5 | 0 | 2 | 5 | 8 | 0 |
| PF3D7_1342000 | 40S ribosomal protein S6 | 0 | 0 | 5 | 0 | 2 | 13 | 57 | 0 |
| PF3D7_1424100 | 60S ribosomal protein L5 | 0 | 0 | 5 | 0 | 0 | 2 | 27 | 0 |
| PF3D7_0821700 | 60S ribosomal protein L22 putative | 0 | 0 | 4 | 0 | 2 | 6 | 15 | 0 |
| PF3D7_0830400 | conserved Plasmodium protein | 0 | 0 | 4 | 0 | 0 | 0 | 6 | 0 |
| PF3D7_1011800 | PRE-binding protein (PREBP) | 0 | 0 | 4 | 0 | 6 | 22 | 4 | 0 |
| PF3D7_1019400 | 60S ribosomal protein L30e | 0 | 0 | 4 | 0 | 0 | 3 | 4 | 0 |
| PF3D7_1136500 | casein kinase 1 | 0 | 0 | 4 | 4 | 4 | 3 | 0 | 0 |
| PF3D7_1142500 | 60S ribosomal protein L28 (RPL28) | 0 | 0 | 4 | 2 | 0 | 7 | 13 | 0 |
| PF3D7_1341200 | 60S ribosomal protein L18 | 0 | 0 | 4 | 0 | 5 | 9 | 23 | 0 |
| PF3D7_1403900 | serine/threonine protein phosphatase CPPED1 | 0 | 0 | 4 | 0 | 0 | 0 | 3 | 0 |
| PF3D7_1419200 | thioredoxin-like protein | 0 | 0 | 4 | 0 | 0 | 0 | 3 | 0 |
| PF3D7_0102900 | aspartate--tRNA ligase | 0 | 0 | 3 | 0 | 0 | 0 | 0 | 0 |
| PF3D7_0306900 | 40S ribosomal protein S23 | 0 | 0 | 3 | 0 | 0 | 5 | 11 | 0 |
| PF3D7_0307200 | 60S ribosomal protein L7 | 0 | 0 | 3 | 0 | 4 | 8 | 30 | 0 |
| PF3D7_0309600 | 60S acidic ribosomal protein P2 (PfP2) | 0 | 0 | 3 | 2 | 3 | 4 | 2 | 0 |
| PF3D7_0402000 | Plasmodium exported protein (PHISTa) | 0 | 0 | 3 | 0 | 0 | 0 | 4 | 0 |
| PF3D7_0702500 | Plasmodium exported protein | 0 | 0 | 3 | 0 | 0 | 4 | 2 | 0 |
| PF3D7_0721600 | 40S ribosomal protein S5 | 0 | 0 | 3 | 0 | 0 | 5 | 14 | 0 |
| PF3D7_0904700 | bacterial histone-like protein (HU) | 0 | 0 | 3 | 0 | 0 | 0 | 0 | 0 |
| PF3D7_0912400 | alkaline phosphatase | 0 | 0 | 3 | 0 | 0 | 2 | 0 | 0 |
| PF3D7_1004000 | 60S ribosomal protein L13 | 0 | 0 | 3 | 0 | 4 | 7 | 18 | 0 |
| PF3D7_1038000 | antigen UB05 | 0 | 0 | 3 | 0 | 0 | 0 | 4 | 0 |
| PF3D7_1105400 | 40S ribosomal protein S4 | 0 | 0 | 3 | 0 | 0 | 6 | 20 | 0 |
| PF3D7_1108400 | casein kinase 2 | 0 | 0 | 3 | 0 | 0 | 5 | 0 | 0 |
| PF3D7_1130100 | 60S ribosomal protein L38 (RPL38) | 0 | 0 | 3 | 0 | 0 | 7 | 18 | 0 |
| PF3D7_1237700 | conserved protein | 0 | 0 | 3 | 0 | 2 | 7 | 18 | 0 |
| PF3D7_1306200 | conserved Plasmodium protein | 0 | 0 | 3 | 0 | 0 | 4 | 0 | 0 |
| PF3D7_1309100 | 60S ribosomal protein L24 | 0 | 0 | 3 | 0 | 0 | 5 | 20 | 0 |
| PF3D7_1317800 | 40S ribosomal protein S19 (RPS19) | 0 | 0 | 3 | 0 | 3 | 4 | 10 | 0 |
| PF3D7_1341300 | 60S ribosomal protein L18-2 | 0 | 0 | 3 | 0 | 0 | 2 | 21 | 0 |
| PF3D7_1346100 | protein transport protein SEC61 subunit alpha (SEC61) | 0 | 0 | 3 | 0 | 0 | 4 | 3 | 0 |
| PF3D7_1358800 | 40S ribosomal protein S15 (RPS15) | 0 | 0 | 3 | 0 | 2 | 8 | 20 | 0 |
| PF3D7_1370300 | membrane associated histidine-rich protein (MAHRP1) | 0 | 0 | 3 | 4 | 7 | 3 | 0 | 0 |
| PF3D7_1424400 | 60S ribosomal protein L7-3 | 0 | 0 | 3 | 0 | 13 | 10 | 35 | 0 |
| PF3D7_0101300 | Pfmc-2TM Maurer's cleft two transmembrane protein | 0 | 0 | 2 | 0 | 0 | 0 | 6 | 2 |
| PF3D7_0107400 | conserved Apicomplexan protein | 0 | 0 | 2 | 0 | 0 | 0 | 3 | 0 |
| PF3D7_0202500 | early transcribed membrane protein 2 (ETRAMP2) | 0 | 0 | 2 | 0 | 4 | 0 | 5 | 3 |
| PF3D7_0507100 | 60S ribosomal protein L4 (RPL4) | 0 | 0 | 2 | 0 | 4 | 19 | 37 | 0 |
| PF3D7_0508000 | 6-cysteine protein (P38) | 0 | 0 | 2 | 0 | 0 | 0 | 0 | 0 |
| PF3D7_0516900 | 60S ribosomal protein L2 (RPL2) | 0 | 0 | 2 | 0 | 0 | 5 | 14 | 0 |
| PF3D7_0517300 | serine/arginine-rich splicing factor 1 (SR1) | 0 | 0 | 2 | 0 | 3 | 8 | 5 | 0 |
| PF3D7_0517400 | FACT complex subunit SPT16 | 0 | 0 | 2 | 0 | 0 | 2 | 0 | 2 |
| PF3D7_0601200 | Pfmc-2TM Maurer's cleft two transmembrane protein | 0 | 0 | 2 | 0 | 0 | 0 | 5 | 2 |
| PF3D7_0618300 | 60S ribosomal protein L27a | 0 | 0 | 2 | 0 | 0 | 7 | 19 | 0 |
| PF3D7_0621200 | pyridoxine biosynthesis protein PDX1 (PDX1) | 0 | 0 | 2 | 0 | 5 | 0 | 2 | 0 |
| PF3D7_0631400 | Pfmc-2TM Maurer's cleft two transmembrane protein | 0 | 0 | 2 | 0 | 0 | 0 | 5 | 2 |
| PF3D7_0710600 | 60S ribosomal protein L34 (RPL34) | 0 | 0 | 2 | 0 | 0 | 2 | 6 | 0 |
| PF3D7_0719600 | 60S ribosomal protein L11a putative | 0 | 0 | 2 | 0 | 0 | 4 | 18 | 0 |
| PF3D7_0801000 | Plasmodium exported protein (PHISTc) | 0 | 0 | 2 | 0 | 0 | 11 | 6 | 0 |
| PF3D7_0815600 | eukaryotic translation initiation factor 3 subunit G putative (EIF3G) | 0 | 0 | 2 | 0 | 2 | 0 | 0 | 0 |
| PF3D7_1026800 | 40S ribosomal protein S2 (RPS2) | 0 | 0 | 2 | 0 | 2 | 4 | 0 | 0 |
| PF3D7_1036900 | conserved Plasmodium protein | 0 | 0 | 2 | 0 | 0 | 14 | 21 | 2 |
| PF3D7_1103100 | 60S acidic ribosomal protein P1 | 0 | 0 | 2 | 0 | 0 | 0 | 0 | 0 |
| PF3D7_1104000 | phenylalanine--tRNA ligase beta subunit | 0 | 0 | 2 | 2 | 2 | 0 | 0 | 0 |
| PF3D7_1106000 | RuvB-like helicase 2 (RUVB2) | 0 | 0 | 2 | 0 | 0 | 2 | 2 | 0 |
| PF3D7_1109900 | 60S ribosomal protein L36 (RPL36) | 0 | 0 | 2 | 0 | 0 | 4 | 11 | 0 |
| PF3D7_1142600 | 60S ribosomal protein L35ae | 0 | 0 | 2 | 0 | 0 | 4 | 12 | 0 |
| PF3D7_1323400 | 60S ribosomal protein L23 (RPL23) | 0 | 0 | 2 | 0 | 2 | 2 | 17 | 0 |
| PF3D7_1325100 | phosphoribosylpyrophosphate synthetase | 0 | 0 | 2 | 0 | 2 | 6 | 0 | 0 |
| PF3D7_1331800 | 60S ribosomal protein L23 | 0 | 0 | 2 | 0 | 0 | 7 | 8 | 0 |
| PF3D7_1407100 | rRNA 2'-O-methyltransferase fibrillarin putative (NOP1) | 0 | 0 | 2 | 0 | 0 | 3 | 11 | 0 |
| PF3D7_1410600 | eukaryotic translation initiation factor 2 subunit gamma | 0 | 0 | 2 | 0 | 0 | 0 | 0 | 2 |
| PF3D7_1459400 | conserved Plasmodium protein | 0 | 0 | 2 | 0 | 0 | 2 | 11 | 0 |
| PF3D7_1462800 | glyceraldehyde-3-phosphate dehydrogenase (GAPDH) | 0 | 0 | 2 | 0 | 7 | 36 | 13 | 6 |
| PF3D7_1218500 | \| dynamin-like protein 3 | 0 | 0 | 10 | 0 | 0 | 0 | 0 | 0 |
| PF3D7_0418200 | \| eukaryotic translation initiation factor 3 subunit M putative | 0 | 0 | 7 | 0 | 0 | 0 | 0 | 0 |
| PF3D7_1473200 | \| DnaJ protein 6 | 0 | 0 | 7 | 0 | 0 | 0 | 0 | 0 |
| PF3D7_0102500 | \| erythrocyte binding antigen-181 | 0 | 0 | 6 | 0 | 0 | 0 | 0 | 0 |
| PF3D7_1108700 | \| heat shock protein J2 | 0 | 0 | 5 | 0 | 0 | 0 | 0 | 0 |
| PF3D7_1314500 | \| transmembrane emp24 domain-containing protein putative | 0 | 0 | 5 | 0 | 0 | 0 | 0 | 0 |
| PF3D7_1362200 | \| RuvB-like helicase 3 | 0 | 0 | 5 | 2 | 0 | 0 | 0 | 0 |
| PF3D7_0422100 | \| transmembrane emp24 domain-containing protein putative | 0 | 0 | 4 | 0 | 0 | 0 | 0 | 0 |
| PF3D7_0811600 | \| conserved protein unknown function | 0 | 0 | 4 | 0 | 0 | 0 | 0 | 0 |
| PF3D7_0823800 | \| DnaJ protein putative | 0 | 0 | 4 | 0 | 0 | 0 | 0 | 0 |
| PF3D7_0829200 | \| prohibitin 1 putative | 0 | 0 | 4 | 0 | 0 | 0 | 0 | 0 |
| PF3D7_0916700 | \| RNA-binding protein musashi putative | 0 | 0 | 4 | 0 | 0 | 0 | 0 | 0 |
| PF3D7_0923000 | \| DNA-directed RNA polymerase II subunit RPB3 4 | 0 | 0 | 4 | 0 | 0 | 0 | 0 | 0 |
| PF3D7_1142800 | \| ATP synthase-associated protein 2 | 0 | 0 | 4 | 0 | 0 | 0 | 0 | 0 |
| PF3D7_0205500 | \| DNA-directed RNA polymerase II 16 kDa subunit putative | 0 | 0 | 3 | 0 | 0 | 0 | 0 | 0 |
| PF3D7_0318100 | \| stomatin-like protein putative | 0 | 0 | 3 | 0 | 0 | 0 | 0 | 0 |
| PF3D7_0413500 | \| phosphoglucomutase-2 | 0 | 0 | 3 | 0 | 0 | 0 | 0 | 0 |
| PF3D7_0611300 | \| ATP synthase-associated protein putative | 0 | 0 | 3 | 0 | 0 | 0 | 0 | 0 |
| PF3D7_1014700 | \| prohibitin 2 2 | 0 | 0 | 3 | 0 | 0 | 0 | 0 | 0 |
| PF3D7_1364800 | \| DNA-directed RNA polymerases I II and III subunit RPABC1 putative | 0 | 0 | 3 | 0 | 0 | 0 | 0 | 0 |
| PF3D7_1432100 | \| voltage-dependent anion-selective channel protein putative | 0 | 0 | 3 | 0 | 0 | 0 | 0 | 0 |
| PF3D7_0212300 | \| eukaryotic peptide chain release factor subunit 1 putative | 0 | 0 | 2 | 0 | 0 | 0 | 0 | 0 |
| PF3D7_0218000 | \| replication factor C subunit 2 putative | 0 | 0 | 2 | 0 | 0 | 0 | 0 | 0 |
| PF3D7_0523600 | \| conserved protein unknown function | 0 | 0 | 2 | 0 | 0 | 0 | 0 | 0 |
| PF3D7_0821800 | \| protein transport protein SEC61 subunit beta putative | 0 | 0 | 2 | 0 | 0 | 0 | 0 | 0 |
| PF3D7_0918000 | \| glideosome-associated protein 50 | 0 | 0 | 2 | 0 | 0 | 0 | 0 | 0 |
| PF3D7_0918300 | \| eukaryotic translation initiation factor 3 subunit F putative | 0 | 0 | 2 | 0 | 0 | 0 | 0 | 0 |
| PF3D7_1013300 | \| conserved Plasmodium protein unknown function | 0 | 0 | 2 | 0 | 0 | 0 | 0 | 0 |
| PF3D7_1014100 | \| merozoite surface protein MSA180 | 0 | 0 | 2 | 0 | 0 | 0 | 0 | 0 |
| PF3D7_1139900 | \| ER membrane protein complex subunit 8 putative | 0 | 0 | 2 | 0 | 0 | 0 | 0 | 0 |
| PF3D7_1149400 | \| Plasmodium exported protein unknown function | 0 | 0 | 2 | 0 | 0 | 0 | 0 | 0 |
| PF3D7_1320600 | \| ras-related protein Rab-11A | 0 | 0 | 2 | 0 | 0 | 0 | 0 | 0 |
| PF3D7_1414800 | \| small nuclear ribonucleoprotein-associated protein B putative | 0 | 0 | 2 | 0 | 0 | 0 | 0 | 0 |
| PF3D7_1417900 | \| ATP synthase-associated protein putative | 0 | 0 | 2 | 0 | 0 | 0 | 0 | 0 |
| PF3D7_1471600 | \| conserved Plasmodium protein unknown function | 0 | 0 | 2 | 0 | 0 | 0 | 0 | 0 |

**Supplementary Table 2**: **MW parameter estimates comparing distribution of ages for children in the highest v. Lowest two antibody tertiles**

| Protein | MW Parameter (95% CI) | MW p-value | MW p-value Adjusted |
| --- | --- | --- | --- |
| DBLγ11_JOSvar266797_FR4_1-2032 | 0.54 (0.47-0.6) | 0.248 | 1.000 |
| DBLζ5_JOSvar222849_FR6_30-1634 | 0.47 (0.41-0.54) | 0.434 | 1.000 |
| DBLζ5_PF3D7_0800200 | 0.5 (0.44-0.57) | 0.944 | 1.000 |
| DBLζ5_HB3var07 | 0.5 (0.44-0.57) | 0.929 | 1.000 |
| PF3D7_0201600_RLP1_frag1 (PHISTb) | 0.51 (0.44-0.57) | 0.837 | 1.000 |
| PF3D7_0201600_RLP1_frag2(PHISTb) | 0.5 (0.43-0.56) | 0.930 | 1.000 |
| Pf3D7_0532300_fragment (PHISTb) | 0.52 (0.45-0.58) | 0.630 | 1.000 |
| PF3D7_0730900_fragment-1(PTP4) | 0.43 (0.37-0.5) | 0.049 | 0.640 |
| PF3D7_0730900_fragagment-2(PTP4) | 0.42 (0.36-0.48) | 0.013 | 0.181 |
| PF3D7_0501400_fragment-1(FIRA) | 0.49 (0.42-0.56) | 0.764 | 1.000 |
| PF3D7_0501400_fragment-2(FIRA) | 0.49 (0.43-0.56) | 0.868 | 1.000 |
| PF3D7_1104400 (Thioredoxin-like mero protein) | 0.41 (0.35-0.48) | 0.008 | 0.120 |
| PF3D7_0201800 (KAHsp40) | 0.41 (0.34-0.47) | 0.005 | 0.077 |
| PF3D7_1015900 (Enolase) | 0.48 (0.41-0.54) | 0.478 | 1.000 |
| PF3D7_0702400 (SIMP1) | 0.53 (0.46-0.59) | 0.395 | 1.000 |

*^a^Abbreviations: CI, confidence interval; MW,* *Mann-Whitney U test;* *DBL, Duffy binding-like (DBL) domain PHISTb,* *Plasmodium helical interspersed subtelomeric proteins; PTP4: Erythrocyte membrane protein 1(EMP1)-trafficking protein 4; FIRA,* *interspersed repeat antigen;* *KAHsp40, knob associated heat shock protein 40; SIMP1,* *small, exported membrane protein 1*
